# Approach to the patient with severe hyperthyroidism-related complications

**DOI:** 10.1210/clinem/dgag112

**Published:** 2026-03-13

**Authors:** Georgios Kostopoulos, Grigoris Effraimidis

**Affiliations:** Department of Endocrinology and Metabolism, Hippokration General Hospital, 54642 Thessaloniki, Greece; Faculty of Medicine, School of Health Sciences, University of Thessaly, 41110 Larissa, Greece; Department of Endocrinology and Metabolic Diseases, Larissa University Hospital, 41110 Larissa, Greece

**Keywords:** hyperthyroidism, thyrotoxicosis, thyrotoxic storm, thyrotoxic periodic paralysis, atrial fibrillation, complications

## Abstract

Hyperthyroidism is a prevalent endocrine disorder that can lead to severe multisystem complications, including atrial fibrillation (AF), thyrotoxic periodic paralysis (TPP), and thyroid storm (TS). This review discusses the pathophysiology, clinical presentation, and management challenges of these complications through illustrative clinical cases. AF is the most common cardiovascular manifestation, with restoration of euthyroidism and rate control being central to treatment. TPP presents with transient muscle weakness due to hypokalemia from intracellular potassium shift, requiring cautious potassium supplementation and hyperthyroidism control. TS is a life-threatening endocrine emergency characterized by multisystem decompensation, necessitating prompt multimodal therapy including antithyroid drugs, beta-blockers, corticosteroids, and supportive care. Thus, early recognition and tailored management remain the cornerstones of successful treatment for these severe complications of hyperthyroidism.

Hyperthyroidism is a common endocrine disorder with a global prevalence of 0.2-2.5% in iodine-sufficient countries, while higher rates have been reported in iodine insufficient countries (1). Thyrotoxicosis (thyroid hormone excess regardless of source or underlying mechanism) and hyperthyroidism (thyroid hormone excess due to overproduction in the thyroid gland) are 2 terms used interchangeably in the literature. The most common causes of hyperthyroidism include Graves' Disease (GD) (thyroid hormone overproduction due to autoantibodies against the thyroid-stimulating hormone [TSH] receptor), toxic multinodular goiter and solitary toxic adenoma (autonomous thyroid hormone secretion due to somatic activating mutations of the TSH receptor, and less often Gsα) (2, 3). Patients with overt hyperthyroidism, defined as having suppressed TSH with elevated free thyroxine (FT4) and/or triiodothyronine (T3) levels, are usually symptomatic, whereas patients with subclinical hyperthyroidism, which constitutes a biochemical diagnosis including low or undetectable TSH with normal free T4 and T3 levels, are oligo- or asymptomatic (2-4). The treatment modalities in hyperthyroidism include antithyroid drugs (ATDs), surgery and radioiodine ablation (RAI). The choice of initial therapy depends on the etiology of hyperthyroidism, patients' characteristics, comorbidities and preferences (2, 3).

Hyperthyroidism can affect multiple major organ systems regardless of cause, and in its more severe forms may precipitate life-threatening complications involving the cardiovascular, skeletal, nervous, and muscular systems, such as atrial fibrillation (AF), thyrotoxic periodic paralysis, and thyroid storm (2, 3). Therefore, prompt recognition and optimal management of severe hyperthyroidism-related complications are of cardinal importance. Nevertheless, there is no robust, high-quality evidence regarding the optimal management of these serious complications and most of the evidence-based recommendations included in recently published consensus statements or guidelines are of low certainty (5, 6). The following illustrative clinical cases will discuss the most common cardiac (AF), the most dramatic neuromuscular (thyrotoxic periodic paralysis) and the most serious (thyroid storm) complication, highlighting the challenges in diagnosis and management.

## Case 1

### Case presentation

A 46-year-old man with unremarkable past medical history presented to the emergency department due to persistent paroxysmal cough and fever. He reported a recent respiratory viral infection. Upon clinical evaluation, irregular heartbeat was detected and electrocardiogram (ECG) confirmed the diagnosis of AF. The patient was admitted to the cardiology department for monitoring and was initiated on bisoprolol 10 mg once daily and apixaban 5 mg twice daily. A routine thyroid function test revealed suppressed TSH (<0.01 mIU/L) and elevated FT4 (2.9 ng/dL [0.7-1.5 ng/dL]) (SI: 37.2 pmol/L [9.0-19.0 pmol/L]) and T3 (15.6 pg/mL [1.6-3.9 pg/mL]) (SI: 24.0 pmol/L [2.4-6.0 pmol/L]) levels and positive thyroid peroxidase antibodies (TPOAbs 1000 IU/L). Electrical cardioversion was planned after normalization of thyroid function and endocrinology consultation was requested. Consequently, initiation of methimazole 20 mg daily was recommended.

### Atrial fibrillation

#### Epidemiology

Atrial fibrillation is the most common cardiovascular manifestation in patients with hyperthyroidism and occasionally may be the first clinical sign of this endocrine condition (7). Recent epidemiological data reported that the estimated prevalence of AF was 5-15% among patients with hyperthyroidism compared to 1-3% in the general population (8-10). Additionally, hyperthyroidism (overt and subclinical) is a risk factor for AF. A recent dose-response meta-analysis of cohort studies reported that patients with overt (HR: 2.35 95% CI [1.07,5.16]) and subclinical hyperthyroidism (HR: 1.7 95% CI [1.11,2.62]) are twice as likely to develop AF compared to euthyroidism (11). Notably, population-based studies also suggest an independent association between AF and high FT4 levels within the normal range (12). On the contrary, higher FT3 levels within the normal range have not consistently been associated with increased AF risk in population studies, although Mendelian randomization data suggest that a higher FT3:FT4 ratio may be genetically linked to increased AF susceptibility (13).

Recent evidence highlights a potential bidirectional relationship of AF and hyperthyroidism. A population registry-based study from Denmark reported that incident hyperthyroidism occurred more frequently in patients with new-onset AF and no underlying thyroid disease compared to the general population (1.3% vs 3.2%) during a follow-up period up to 13 years (14). However, these findings should be interpreted acknowledging the limitations of the study, namely the high risk for detection and protopathic bias. Moreover, no clear pathophysiological mechanism has been established to explain how AF itself could induce hyperthyroidism.

#### Risk factors

Atrial fibrillation typically develops early in the course of hyperthyroidism, usually within 3 months from diagnosis; however, cases with late-onset AF have also been reported even after achieving euthyroidism (15). The risk factors of AF in patients with hyperthyroidism do not differ from those in patients without hyperthyroidism, including older age, male sex, ischemic heart disease, heart failure, and valvular disease (15, 16). No prognostic models have been developed or validated specifically for incident AF prediction in patients with hyperthyroidism; the C_2_HEST model (coronary artery disease or chronic obstructive pulmonary disease [1 point each], hypertension [1 point], elderly [age ≥ 75 years, 2 points], systolic heart failure [2 points], and thyroid disease [hyperthyroidism,1 point]), which was developed for AF prediction in the general population, may be useful in the setting of hyperthyroidism (17). On the basis of the above, the American College of Cardiology (ACC), the American Heart Association (AHA), and the European Society of Cardiology (ESC) endorse routine thyroid function testing for all patients with new-onset AF (18, 19). In parallel, the American Thyroid Association (ATA) and the European Thyroid Association (ETA) highlight the need for cardiovascular evaluation—including electrocardiography (ECG), Holter ECG, and Doppler echocardiography—in patients with hyperthyroidism who are older (typically ≥65 years) or have cardiovascular comorbidities, and in selected patients with grade 2 subclinical hyperthyroidism (TSH <0.1 mIU/L), in whom the risks of AF, heart failure, and other cardiac events are increased (5, 6, 20).

#### Management

High-quality evidence regarding the management of AF in patients with hyperthyroidism is lacking, as patients with thyroid dysfunction were excluded from the majority of pivotal randomized controlled trials evaluating potential therapeutic interventions for AF (21). An overwhelming body of evidence supports the AF Better Care (ABC) pathway for a multifactorial, personalized approach to risk reduction and treatment selection in patients with AF. The ABC pathway is a 3-pillar approach: (1) A for avoiding stroke (anticoagulation), (2) B for better symptom management (rate and rhythm control), and (3) C for cardiovascular risk and comorbidities management. In light of the above, the ABC approach may be adapted with certain modifications to effectively manage patients with hyperthyroidism-related AF (22, 23).

##### Restore euthyroidism

Hyperthyroidism is a reversible cause of AF and thus, prompt restoration of thyroid function is the cornerstone of management. Approximately two-thirds of patients with hyperthyroidism experience spontaneous return to sinus rhythm after or even before achieving euthyroidism, highlighting the importance of controlling thyroid status, especially in patients with overt hyperthyroidism (24, 25). In cases with subclinical hyperthyroidism, treatment is recommended in patients with suppressed TSH (<0.1 mIU/L), older age (>65) or comorbidities (cardiovascular disease or osteoporosis) (4, 5). Evidence regarding the optimal TSH target is inconclusive and targeting for a TSH within the age-specific normal reference range is well grounded (7). Notwithstanding the latter, there remains clinical uncertainty as to whether treatment of subclinical hyperthyroidism leads to AF risk reduction.

ATDs, namely methimazole (MMI), carbimazole (CBZ, a prodrug of MMI), and propylthiouracil (PTU), are considered first-line options for the management of hyperthyroidism in short-term, as they are highly efficacious and offer rapid restoration of thyroid function. These drugs inhibit thyroid hormone synthesis; MMI is generally preferred due to a more favorable safety profile (ie, increased risk of hepatotoxicity and fulminant hepatitis with PTU), while PTU is recommended during the 1st trimester of pregnancy due to lower teratogenic effects. Observational data suggest that definitive therapies (surgery and RAI) may be more effective in mitigating AF risk, offering long-term protection (26-28).

##### “A” avoid stroke (anticoagulation)

Hyperthyroidism is associated with a hypercoagulable state, characterized by elevated levels of various factors implicated in coagulation and fibrinolysis, such as plasma fibrinogen, von Willebrand factor, and factor X (29). However, observational studies fail to demonstrate a robust association of hyperthyroidism and increased risk for ischemic stroke and systemic embolism. This uncertainty is also reflected in the 2023 ACC/AHA/American College of Chest Physicians (ACCP)/Heart Rhythm Society (HRS) guideline, which concludes that the role of hyperthyroidism as an independent stroke risk factor remains unclear (18). Two large population-based studies from France and Korea suggested an independent association between hyperthyroidism and ischemic stroke and systemic embolism (30, 31). On the contrary, evidence from the Swedish Atrial Fibrillation Cohort Study suggested that hyperthyroidism does not constitute an independent risk factor for stroke (32). Another retrospective cohort study from Taiwan reported that patients with hyperthyroidism-related AF had lower risk for stroke and systemic thromboembolism compared to nonthyroid AF (33). Inconsistencies among studies may be due to different study designs, methodology, participants baseline characteristics, and treatment with anticoagulation.

The necessity of thrombosis prevention in hyperthyroidism-related AF has not yet been clearly established. There is paucity of randomized trials on the potential benefits and harms of long-term anticoagulation therapy in hyperthyroidism-related AF. Despite the high rate of spontaneous reversibility to sinus rhythm once euthyroidism is controlled, patients with hyperthyroidism-related AF may be at increased risk for thromboembolic events during thyrotoxic state.

Current guidelines do not include specific recommendations for anticoagulation initiation in hyperthyroid patients and advocate the application of CHA_2_DS_2_-VASc and HAS-BLED scores to guide anticoagulation decisions, as in nonthyroid AF (18, 19, 34). The CHA_2_DS_2_-VASc score (1 point for the presence of congestive heart failure, hypertension, age between 65 and 74 years, female sex, diabetes, and vascular disease, 2 points for age older than 75 years and previous stroke) is a point scale system estimating stroke risk for nonvalvular AF. CHA_2_DS_2_-VASc can accurately identify low- and high-cardioembolic risk patients and thus prioritize thromboembolic prevention in those with higher risk (≥1 men or ≥2 women). On the contrary, HAS-BLED score (hypertension >160 mmHg, abnormal renal function; creatinine >2.26 mg/dL, abnormal liver function; cirrhosis or bilirubin >2× normal with transaminases/alkaline phosphatase >3× normal, stroke, bleeding or predisposition to bleeding, labile INR, age >65, medications predisposing to bleeding, alcohol use) is widely applied for bleeding risk assessment in patients with AF (score ≥3 high bleeding risk). It should be underlined, though, that these scores were not specifically developed or validated in hyperthyroid patients. External validation studies in populations with hyperthyroidism suggest that they may either overestimate or underestimate the actual risk of stroke or bleeding (18, 19, 34).

A meta-analysis reported that oral anticoagulation in high-risk patients (CHA_2_DS_2_-VASc score ≥1) with hyperthyroidism and AF was effective, leading to a 3% risk reduction of ischemic stroke and systemic embolism compared to no anticoagulation (35). Furthermore, data from the ARISTOTLE trial highlight that patients with AF and hyperthyroidism treated with oral anticoagulation had similar clinical outcomes to those without thyroid disease (36). Warfarin, a commonly used vitamin K antagonist, was highly efficacious in reducing stroke risk by 70% in patients with nonself-limiting hyperthyroidism-related AF and CHA_2_DS_2_-VASc score ≥1 (37). However, patients with hyperthyroidism may require lower doses of warfarin to maintain INR within the therapeutic range, as hyperthyroidism enhances the degradation of vitamin K-related factors (7). Additionally, the evidence for the efficacy of newer direct oral anticoagulants (DOACs) in hyperthyroidism is insufficient. A post hoc analysis of the ARISTOTLE trial found no significant difference in efficacy and bleeding risk between apixaban and warfarin (36). Conversely, a retrospective cohort study from Taiwan demonstrated a lower risk of bleeding among patients receiving DOACs compared with warfarin (HR 0.65, 95% CI 0.44-0.96), with similar efficacy between the 2 treatment groups (38).

##### “B” better symptom control (rate vs rhythm control)

Rate control has a primary role in the setting of hyperthyroidism-related AF, whereas rhythm control is reserved for cases of persistent AF. Older age, longer AF duration, and uncontrolled hyperthyroidism are the main determinants of persistence of AF (21, 34, 39).

In patients with hyperthyroidism-related AF, nonselective beta-blockers (propranolol preferred) are recommended as first-line therapy for ventricular rate control and relief of adrenergic hyperthyroid symptoms; dosing can be initiated at 10-40 mg orally every 6-8 hours and titrated to a resting heart rate <110 beats per minute with adequate symptom control.

Furthermore, treatment with beta-blockers may prevent subsequent tachycardia-induced or dilated cardiomyopathy. Of note, propranolol may also inhibit the peripheral conversion of T4 to T3; however, this effect may be of limited clinical value, as propranolol reduces T3 in much higher doses than those applied in clinical practice. Caution is warranted in patients with heart failure and airway diseases (asthma and chronic obstructive pulmonary disease), in whom beta-blockers may precipitate decompensation. In such cases, beta-blockers should be used judiciously and under close monitoring in patients with heart failure, whereas non-beta-blocking agents such as calcium-channel blockers or digoxin can be used for rate control in patients with airway disease. In pregnancy, cardioselective beta-blockers are generally preferred, and the lowest effective dose of metoprolol should be used for the shortest possible duration (5, 6).

Rhythm control has a secondary role, as AF is self-limiting in most patients with concurrent hyperthyroidism, and is reserved for patients with persistent hyperthyroidism-related AF, defined as AF duration of greater than 4-6 months after achieving euthyroidism (7, 34). Rhythm control includes pharmacological (Class IA, IC and III antiarrhythmic agents, caution with amiodarone due to the potential risk of amiodarone-induced thyroid dysfunction) and electrical cardioversion. Prior to cardioversion, patients should maintain euthyroidism for at least 4 months and receive a minimum of 3 weeks of anticoagulation, unless transesophageal echocardiography excludes thrombosis within the left atrium (7, 34).

##### “C” (cardiovascular disease and comorbidities)

As current guidelines recommend, optimal control of comorbidities and modifiable risk factors, together with lifestyle optimization and appropriate treatment, is crucial for minimizing residual cardiovascular risk (7).

**Figure 1:**
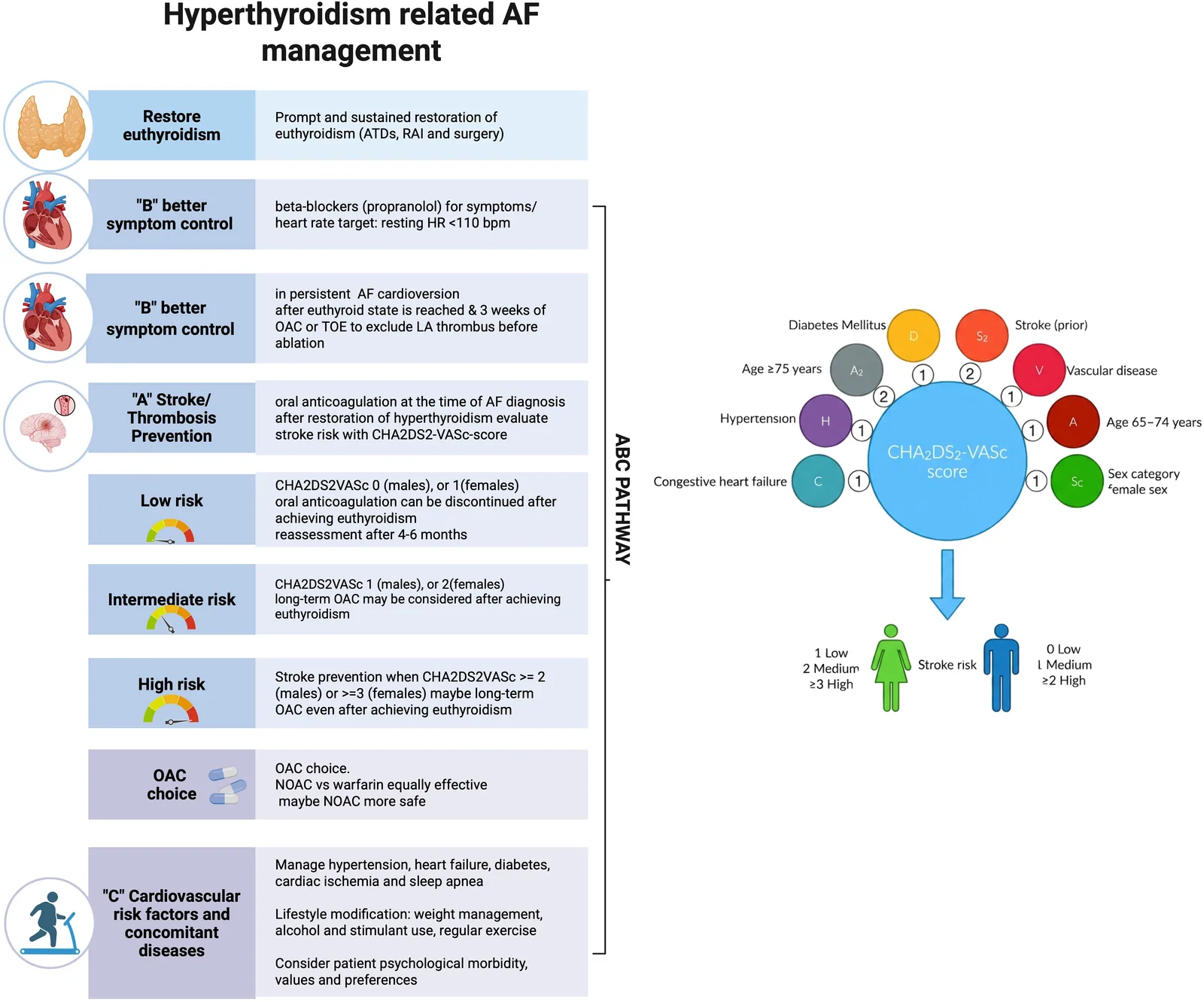
Algorithm for the management of atrial fibrillation in hyperthyroidism (AF, atrial fibrillation; ATDs, antithyroid drugs; HR, heart rate; LA, left atrial; NOAC, novel oral anticoagulant; OAC, oral anticoagulant; RAI, radioiodine ablation; TOE, transoesophageal echocardiogram).

Overall, restoring euthyroidism and rate control are of paramount importance when treating patients with hyperthyroidism-related AF. Oral anticoagulation may be initiated at the time AF is diagnosed, regardless of baseline CHA_2_DS_2_-VASc score, to provide protection during the phase of active thyrotoxicosis and early rhythm instability. After achieving euthyroidism, anticoagulation continuation should be individualized using standard stroke risk stratification tools, while acknowledging their limitations, the often transient course of thyrotoxic AF, and the paucity of robust evidence. A proposed algorithm is in Fig. 1.

### Case discussion

Within 15 days of methimazole initiation, resolution of hyperthyroidism and subsequent return to sinus rhythm were documented. During follow-up, bisoprolol and apixaban were discontinued, and methimazole was continued with the dose titrated according to serial thyroid function tests, on which he has remained euthyroid. Overall, this case is consistent with autoimmune hyperthyroidism complicated by new-onset AF following a recent viral infection. Given a CHA_2_DS_2_-VASc score of 0, anticoagulation was deemed unnecessary after euthyroidism was achieved, considering the patient's low risk of stroke or systemic embolism and the rapid resolution of AF after restoration of euthyroidism. This case highlights that restoration of euthyroidism and appropriate rate control are the key principles in management.

## Case 2

### Case presentation

A 39-year-old Caucasian male presented to the emergency department with sudden-onset muscle weakness, fatigue, and vomiting. He was hemodynamically stable and afebrile (body temperature 36.3^o^C [97.3^o^F]) with oxygen saturation (SpO_2_) of 98%, heart rate of 93 bpm, blood pressure of 150/60 mmHg. Neurological examination revealed flaccid, symmetrical proximal muscle weakness of the upper (3/5 right and 2/5 left) and lower limbs (2/5 both) with preserved sensory function, diminished to absent deep tendon reflexes, and normal plantar reflexes. Laboratory evaluation showed marked hypokalemia (serum potassium 1.56 mmol/L), while arterial blood gas analysis revealed normal acid-base status. Due to abnormal ECG findings, he was initially admitted to the coronary care unit for monitoring and was initiated with intravenous potassium and magnesium supplementation. Further evaluation for the underlying cause of hypokalemia revealed suppressed TSH (<0.008 mIU/L), elevated FT4 (3.1 ng/dL [0.7-1.5 ng/dL]) (SI: 39.5 pmol/L [9.0-19.0 pmol/L]), and FT3 (9.05 pg/mL [1.6-3.9 pg/mL]) (SI: 14.0 pmol/L [2.4-6.0 pmol/L]). Subsequent ultrasound examination showed a significantly enlarged thyroid gland. The patient was treated with methimazole 20 mg daily and propranolol 20 mg 3 times daily.

### Thyrotoxic periodic paralysis

#### Epidemiology

TPP is a rare, potentially fatal, but reversible manifestation of thyrotoxicosis. TPP belongs to a group of neuromuscular disorders called channelopathies and is characterized by transient episodes of muscle weakness associated with hypokalemia and thyrotoxicosis (40). TPP mainly affects men of Asian origin (male-to-female ratio 20:1) in the second to fourth decade of life, with an estimated incidence of 2% compared to 0.2% in non-Asian thyrotoxic patients (41, 42). However, cases in non-Asian populations, including African American, Native American, and Hispanic individuals and Caucasian patients such as ours, are well documented (43). Emerging epidemiological data demonstrate a significantly higher risk among Hispanic populations, indicating a TPP prevalence in Hispanic patients with hyperthyroidism of approximately 0.5%, noticeably higher than the traditional 0.1% to 0.2% estimate for non-Asian cohorts (44, 45). Because of the historical misconception that TPP is exclusively an Asian-predominant condition, there is a critical concern for under-diagnosis and delayed clinical intervention in Central American and broader Hispanic populations. Moreover, increasing global migration means clinicians should remain alert to this diagnosis irrespective of ethnic background. GD is the main cause of the disorder, but TPP has also been described in patients with other forms of thyrotoxicosis, such as toxic multinodular goiter and subacute thyroiditis (46, 47).

#### Pathophysiology and genetics

The hallmark of the disease is hypokalemia due to intracellular shift of potassium rather than a true potassium deficit. Nevertheless, the exact pathophysiologic mechanism of TPP has not been fully elucidated yet. Studies in rats, cultured myotubes, and human muscle biopsies all show that excess thyroid hormone consistently increases both the abundance and activity of Na^+^/K^+^-ATPase via genomic and nongenomic pathways (48, 49). Adrenergic activity, which is increased in thyrotoxicosis, further stimulates Na^+^/K^+^-ATPase. This enhanced pump activity leads to paradoxical membrane depolarization, Na^+^ channel inactivation and subsequently muscle inexcitability and paralysis. Other hormones that may sensitize Na^+^/K^+^-ATPase include insulin, catecholamines and androgens (50-52). This may explain why TPP attacks occur after events stimulating insulin or catecholamine secretion (53) (such as high-carbohydrate ingestion, strenuous exercise, alcohol ingestion, cold exposure, emotional and somatic stress, and drugs [ie, corticosteroids and epinephrine]) (54), and why men are more often affected, despite female preponderance in thyroid diseases (55).

If thyroid hormone excess and common triggers such as high-carbohydrate loads or strenuous exercise are sufficient to drive the characteristic intracellular potassium shift, why do only a minority of hyperthyroid patients with a particular ethnic background exposed to these stimuli develop TPP? It has been hypothesized that TPP may have a genetic basis. Emerging data highlight loss-of-function mutations of KCNJ18 encoding the Kir2.6 channel may contribute to TPP development (56, 57). In fact, mutations of KCNJ18 were identified in one-third of patients with TPP in Caucasians and Latin Americans. Furthermore, polymorphisms in Kir2.1, CACNAIS, SCN4A, and ABCC8 have been reported (56, 57). A more recent case-control genome-wide association study in a Chinese Han population identified 5 genetic loci; 3 TPP-specific (DCHS2 on 4q31.3; C11orf67 on 11q14.1; 17q24.3 near KCNJ2) and 2 shared with GD (MHC and Xq21.1), suggesting that TPP is not just a complication but it may constitute a distinct molecular subtype of GD (58).

#### Clinical manifestations and diagnosis

TPP attacks occur suddenly, typically during the night or early in the morning, and can range from mild muscle weakness to flaccid paralysis. Initially, the proximal muscles of the lower limbs are affected, but TPP may progress to quadriplegia and generalized weakness. Most of the time, TPP episodes are transient (may last up to 72 hours from symptom onset) with complete recovery (59). Rarely, more clinically severe phenotypes may also develop, such as the occurrence of fatal cardiac arrhythmias and respiratory failure due to involvement of respiratory muscles and diaphragm (46, 55, 59). During physical evaluation, hypotonia and hypo or areflexia may be documented, while sensory and autonomic functions are preserved.

Laboratory evaluation reveals hypokalemia with low urinary potassium excretion and thyrotoxicosis, which establishes the diagnosis of TPP (3). By definition, a diagnosis of TPP requires biochemical confirmation of thyrotoxicosis, as TPP episodes occur exclusively in the thyrotoxic state and not during euthyroidism. Sometimes, potassium levels may be within the normal range, especially during recovery. Other biochemical abnormalities that may be documented are low urinary potassium excretion and low serum phosphate and magnesium levels.

#### Management

Due to the rarity of the disease, evidence regarding management is of low quality (case reports, retrospective cohort studies).

Prompt recognition and management of TPP is essential, as TPP is a potentially life-threatening disorder. The mainstays of management lie along 2 axes: first, potassium supplementation; and second, control of the underlying hyperthyroid state. Close ECG and electrolyte monitoring is necessary (60). Potassium is administered intravenously (∼10 mEq/h). Avoiding dextrose-containing solutions is essential, as they may worsen hypokalemia and paralysis due to further insulin-mediated activation of Na+/K+-ATPase. It should be underlined though, that potassium should be repleted with caution due to the risk of rebound hyperkalemia during aggressive potassium replacement. Furthermore, coadministration of magnesium may also be applied. In case of life-threatening arrhythmias or respiratory failure, much higher doses of intravenous potassium may be administered (up to 40mEq/h) until patients' stabilization. Moreover, nonselective beta-blockers have a central role in the management of TPP, as they inhibit β_2_-adrenergic stimulation of Na^+^/K^+^-ATPase and prevent intracellular potassium shift (61).

**Figure 2:**
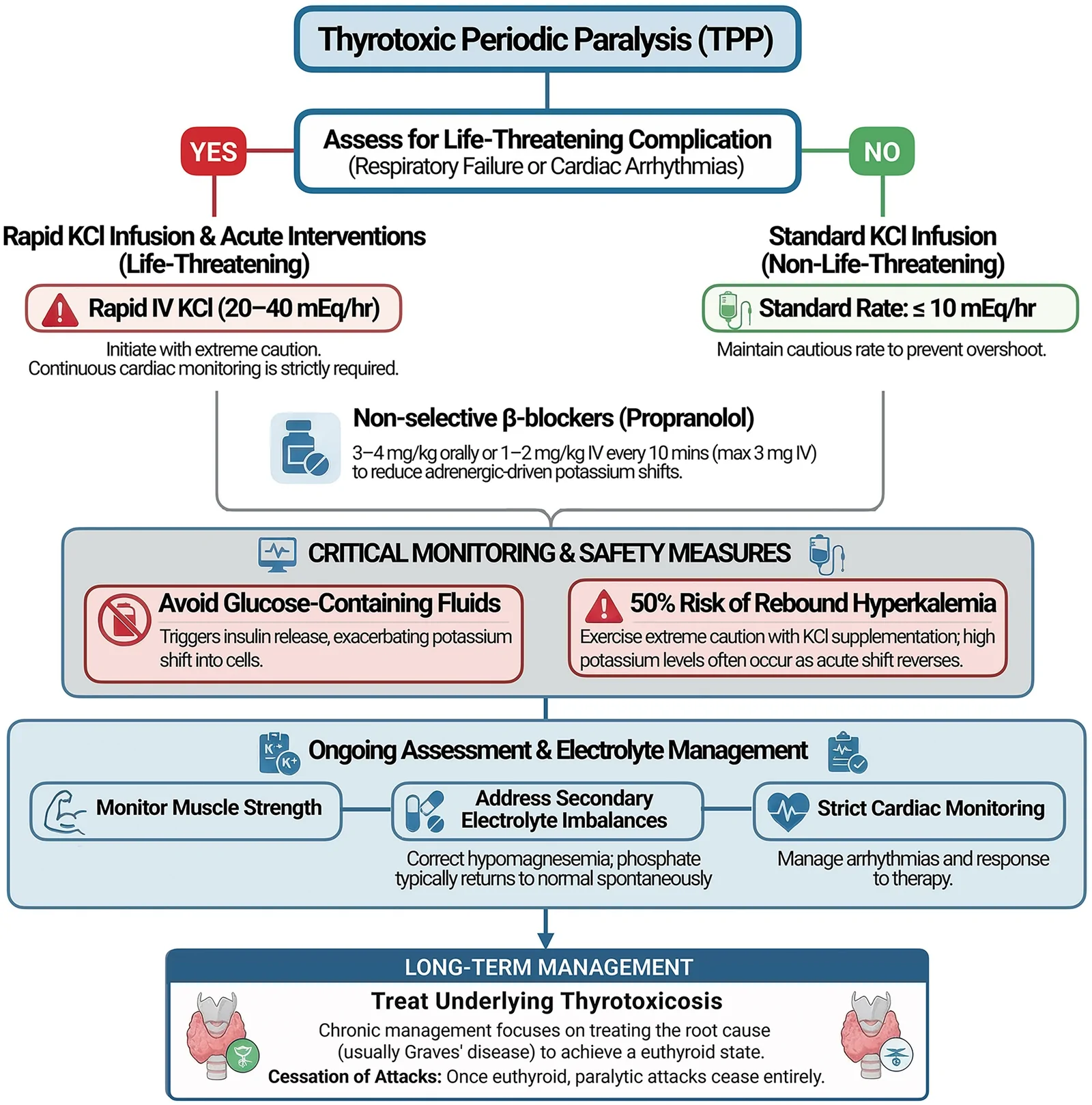
Overview of the thyrotoxic periodic paralysis (TPP) management (IV, intravenous; KCl, potassium chloride).

Treatment of the underlying hyperthyroidism is also of cardinal importance, as TPP has excellent prognosis with low risk of recurrence when euthyroidism is achieved and maintained. However, no specific recommendations exist for the management of hyperthyroidism in TPP per se, and therefore, treatment should follow current ATA and ETA guidelines (3, 5, 6). During the acute thyrotoxic phase, ATDs and beta-blockers are the preferred agents (60). In case of contraindications, ie, in ATDs agranulocytosis, other therapies, including lithium and corticosteroids, may be applied (62). Definitive treatments (RAI and surgery) are generally favored for long-term management. A retrospective cohort study including patients with GD from Asia supports this notion, as it was reported that RAI was associated with a lower risk of recurrence of TPP compared to ATDs (63). Patients should also avoid triggers, such as high-carbohydrate ingestion, exercise, or drugs until euthyroidism is restored. Ηigh-dose intravenous glucocorticoid therapies such as methylprednisolone used for thyroid eye disease, or hydrocortisone utilized in the management of thyroid storm have been reported to precipitate TPP in uncontrolled thyrotoxic patients (64-66). In these individuals, in-patient monitoring with serial potassium and ECG for 24 hours after the first glucocorticoid infusion may be considered in poorly controlled or high-risk patients. Prophylactic potassium supplementation in the euthyroid state is not recommended (3, 60). TPP's prognosis is excellent when thyrotoxicosis is appropriately treated. An overview of TPP management is presented in Fig. 2.

### Case discussion

The patient's symptoms improved within hours after potassium and magnesium replacement, and he was transferred to the Internal Medicine Department. Further evaluation of the etiology of hyperthyroidism revealed positive TRAb and TPOAb and while thyroid ultrasound demonstrated a markedly enlarged thyroid gland, findings consistent with GD. Although no clear precipitating factor was identified, the typical clinical presentation and the combination of hypokalemia and thyrotoxicosis were highly suggestive of thyrotoxic periodic paralysis.

The patient's hyperthyroidism was initially managed with methimazole 20 mg once daily and propranolol 20 mg 3 times daily, which rapidly controlled adrenergic symptoms and led to progressive normalization of thyroid hormone levels over the following weeks. Methimazole was subsequently titrated according to thyroid function tests with the aim of achieving and maintaining euthyroidism, given that recurrence of TPP is closely linked to persistent or recurrent thyrotoxicosis. Once euthyroid, the patient experienced no further episodes of weakness during follow-up, and he was counseled that definitive control of GD is essential to minimize the long-term risk of recurrent TPP. He was also advised to avoid potential triggers, including heavy carbohydrate loads, alcohol consumption, and strenuous physical exertion, until his thyroid function was fully normalized. This case highlights the reversibility of the condition and its excellent clinical outcome when managed appropriately.

## Case 3

### Case presentation

A 47-year-old female presented in a comatose state with tachycardia, hypertension, and hyperpyrexia, following a 5-day history of viral respiratory infection. Upon arrival at the hospital, tracheal intubation was performed and the patient was admitted to the intensive care unit for suspected meningoencephalitis. Her clinical course rapidly deteriorated with hemodynamic instability requiring noradrenaline, along with persistent hyperpyrexia and tachyarrhythmia. Cerebrospinal fluid analysis was unremarkable except for elevated lactate. Thyroid evaluation revealed severe thyrotoxicosis with undetectable TSH (<0.005 mIU/mL) and elevated FT4 (4.05 ng/dL, 52.1 pmol/L; reference range 0.82-1.77 ng/dL, 10.6-22.8 pmol/L) and FT3 (18.1 pg/mL, 27.8 pmol/L; reference range 2.0-4.4 ng/dL, 3.1-6.8 pmol/L) and a large goiter with bruit, suggestive of GD. The Burch–Wartofsky score was 85, highly supportive of the diagnosis of thyrotoxic storm, likely precipitated by acute SARS-CoV-2 infection, as confirmed by a positive rapid nasal swab.

### Thyroid storm

#### Epidemiology

Thyrotoxic storm (TS) is a rare, life-threatening state of decompensated thyrotoxicosis and severe end-organ dysfunction. The estimated incidence of TS ranges from 0.20 to 1.4/100 000 persons hospitalized among different geographical regions due to inconsistencies in TS definition (67). Despite prompt recognition and aggressive management, mortality remains high (10-25%) (2). Recent epidemiological data from Japan highlight that the prognosis of patients with TS has significantly improved over the last decade. Specifically, the overall in-hospital mortality rate has declined to 5.4%, which is approximately half of the 10.7% mortality rate reported in previous nationwide surveys (68). In the U.S., analysis of hospitalized patients with TS between 2004 and 2013 revealed an in-hospital mortality ranging from 1.2% to 3.6% (69). In a more recent study covering 2016 to 2020, the reported mortality in patients admitted with TS increased over time (4.15% in 2020 vs 0.62% in 2016), although this association was not statistically significant, while overall hospitalizations for thyrotoxicosis declined (70). This pattern likely reflects earlier recognition of thyrotoxicosis due to increased clinical awareness, prompt intervention before progression to severe thyrotoxicosis and a shift toward hospitalizing the most severely ill patients, rather than a true worsening of prognosis. In addition to clinical and comorbidity-related factors, such as young age, urgency of admission, smoking status, burden of organ dysfunction, and coexisting congestive heart failure or chronic liver disease, socioeconomic status and access to care may substantially influence both the likelihood of hospitalization and the severity at presentation in patients with thyrotoxicosis (70, 71). The most frequent causes of death were cardiac failure, arrhythmias, respiratory failure, and shock (68, 70).

#### Precipitating factors

Thyrotoxic storm occurs in the setting of long-standing, untreated or undertreated hyperthyroidism. Nearly 90% of the patients with TS have underlying GD, but patients with other causes of thyrotoxicosis have also been described in the literature (67, 72, 73). Additionally, TS may also manifest after a sudden trigger factor, which surpasses the compensatory mechanisms. However, up to 43% of cases have no identifiable trigger (68). Common precipitating factors may involve ATDs nonadherence, infection, trauma, thyroid or nonthyroid surgery, iodine load and parturition (68). It has been hypothesized that TS is the result of a rapid increase of thyroid hormones and sensitivity to catecholamines (67).

#### Clinical manifestations

Patients with TS typically exhibit an exaggeration of the usual symptoms of hyperthyroidism including multiple systems, such as cardiovascular, neurologic, and gastrointestinal. However, TS can also occur in elderly patients with masked or “apathetic” thyrotoxicosis, in whom hyperthyroidism is present but classic symptoms are minimal or absent (3, 74, 75). In a retrospective cohort of hospitalized patients with thyrotoxicosis, the TS patients were more likely to present with tachycardia (RR ≈ 10), altered mentation (RR ≈ 9), fever (RR ≈ 6), and an identifiable precipitating event (RR ≈ 4) than those with uncomplicated thyrotoxicosis (76).

#### Laboratory evaluation and diagnosis

Biochemical evidence of hyperthyroidism is highly suggestive of TS, but clinical judgment is necessary, as the degree of thyroid hormone levels is not diagnostic of TS, per se (67, 72, 76). Total and free thyroid hormone concentrations are usually raised, yet there is no specific threshold that establishes TS, and levels may overlap with those seen in uncomplicated thyrotoxicosis; moreover, serum T3 can be normal in patients who are severely or protractedly ill because of non-thyroidal illness (76). Accordingly, appropriate therapy should be initiated promptly when a TS is suspected rather than delayed until thyroid function test results are available. Other laboratory findings are common but nonspecific including leukocytosis, mild hypercalcemia and hyperglycemia and abnormal liver function tests and evaluation should be tailored to the clinical context (76).

**Figure 3:**
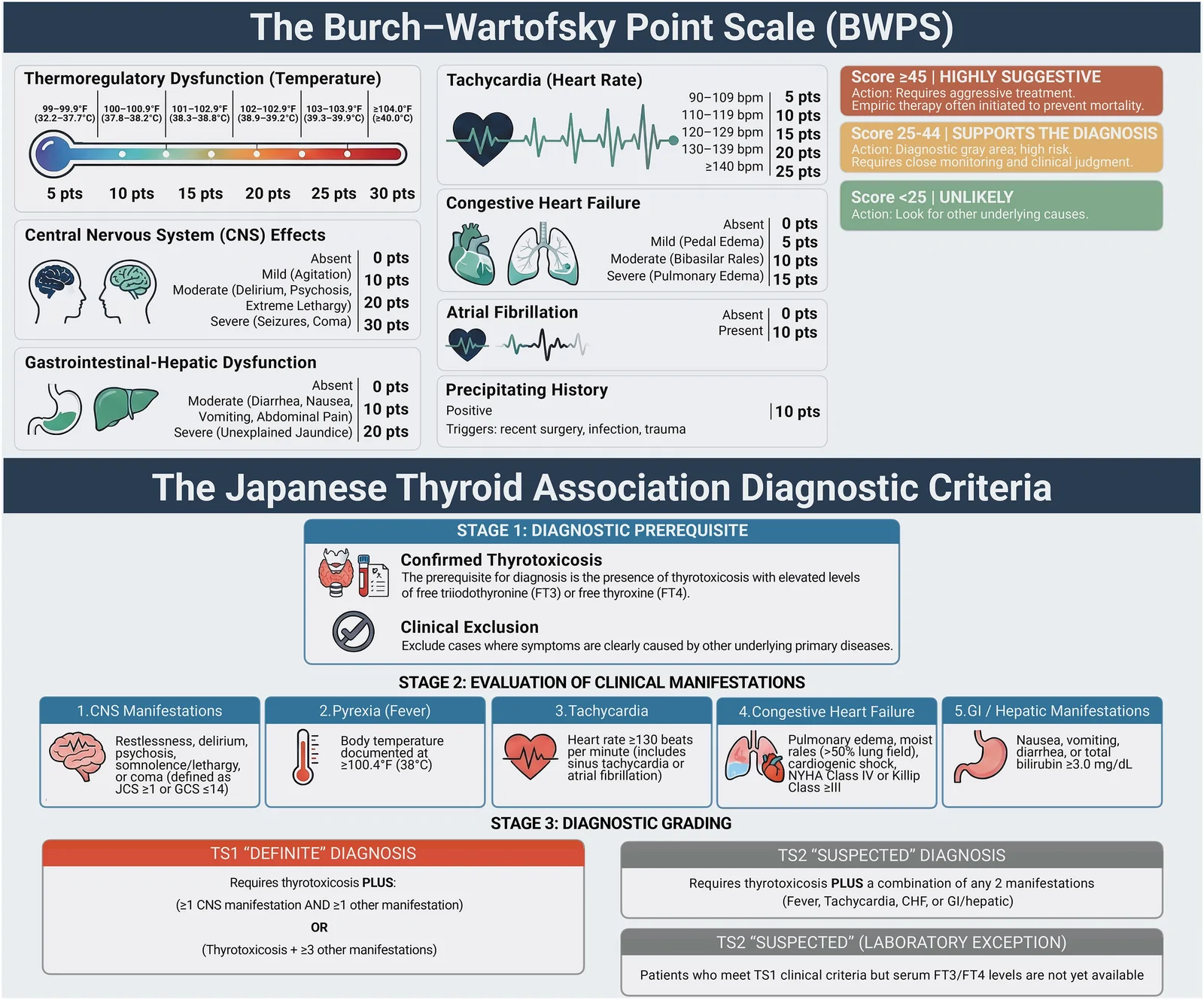
Diagnostic criteria for thyroid storm (TS)—Burch–Wartofsky point scale vs Japanese Thyroid Association Criteria (CHF, congestive heart failure; GI, gastrointestinal).

Diagnosis of TS is based on clinical grounds and a history of pre-existing hyperthyroidism, enlarged goiter, or thyroid eye disease often raises the level of suspicion. There are no universally standardized diagnostic criteria of TS (2). To address this gap 2 clinical tools for TS diagnosis, which incorporate clinical variables, have been developed. In 1993, Burch and Wartofsky developed a risk score (BWPS) to evaluate the likelihood of TS (77, 78). A score greater than 45 is highly supportive of TS, 25 to 44 of impending TS and lower than 25 makes the diagnosis of TS less likely. The second scoring system was introduced in 2012 from the Japan Thyroid Association (JTA). Unlike BWPS, the JTA criteria require documented biochemical thyrotoxicosis as an entry condition and do not rely on a numerical score, but instead emphasize the presence of systemic decompensation and either classifies TS as “definite” or “suspected” (79) (Fig. 3).

Recent studies comparing the performance of the BWPS and the JTA scores suggest that BWPS tends to be more sensitive but less specific, whereas the JTA criteria are more specific but may miss some clinically diagnosed cases. Consequently, neither scoring system should replace expert clinical judgment; instead, both should be used as adjunctive tools to support, rather than dictate, clinical decision-making when ruling in or ruling out thyroid storm (68, 76).

#### Management

**Figure 4:**
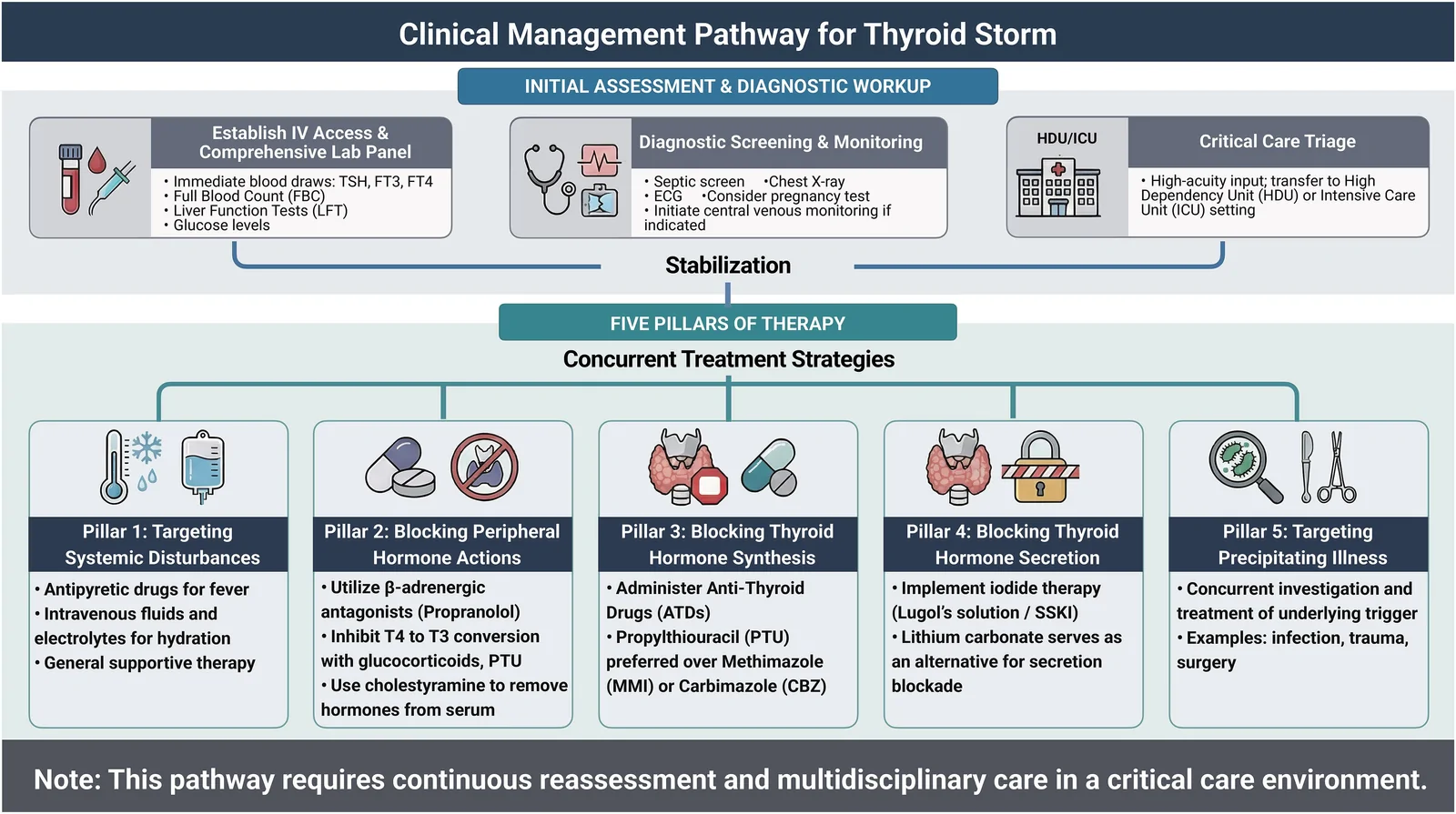
Multimodal management of thyroid storm (TS) (ATDs, antithyroid drugs; CBZ, carbimazole; ECG, electrocardiogram; fT3, free triiodothyronine; fT4, free thyroxine; HDU, high dependency unit; ICU, intensive care unit; IV, intravenous; MMI, methimazole; PTU, propylthiouracil; SSKI, saturated solution of potassium iodide; T3, triiodothyronine; T4, thyroxine; TSH, thyroid stimulating hormone; XRAY, X-ray).

TS is an endocrine emergency, and immediate treatment initiation is recommended when it is suspected, even before laboratory confirmation (67). Once again, due to the scarcity of disease there is paucity of clinical trials for the management of TS and most evidence-based recommendations are based on good clinical practice and are of low grade in evidence (5, 6). The management of TS typically involves a 5- pillar approach (Fig. 4):

##### 1. Supportive measures and hemodynamic stabilization

A multidisciplinary team approach is essential. Patients should be managed in an intensive care unit (ICU) setting for intensive monitoring and treatment. Initial management includes appropriate supportive care: cooling measures, acetaminophen/paracetamol (avoiding aspirin, as it can increase circulating thyroid hormone levels by displacing them from protein-binding sites), and appropriate intravenous fluids with electrolyte correction. Close monitoring of glucose levels is required, as patients are at risk of hypoglycemia. Nutritional support, including vitamin replacement (eg, thiamine), must be considered. Chlorpromazine 50-100 mg IM can be considered when sedation is required, which will also reduce temperature. Clinicians should maintain a high index of suspicion for an increased risk of requiring respiratory support (67, 80).

##### 2. Addressing the peripheral actions of thyroid hormones

###### Beta-blockade

Propranolol 60-80 mg orally every 4 hours is recommended. These high doses also have the potential advantage of reducing T4 to T3 conversion. Intravenous administration (1-3 mg at a maximum rate of 1 mg/min intravenous, repeated only once after 2 minutes, with infusion at 2-3 mg/hour) is an option if the patient is unstable or unable to take medication orally due to vomiting or agitation. Many clinicians use intravenous esmolol instead of propranolol due to its short half-life, allowing rapid discontinuation if adverse outcomes occur (loading dose: 250-500 mcg/kg over 1 minute; maintenance: 0-200 mcg/kg/min) (6, 79, 81). Beta-blockers should be used with caution in patients with systolic heart failure as they can potentially precipitate cardiovascular collapse. Invasive monitoring in patients with a history of or suspected congestive heart failure should be considered. Diltiazem (60-90 mg orally every 6 hours, or intravenous) may be beneficial if beta-blockers are contraindicated (eg, asthma, cardiac decompensation) (67, 79).

###### Corticosteroids

Intravenous hydrocortisone 100 mg loading dose, then 50 mg 4 times a day. High-dose steroid therapy decreases conversion of T4 to T3. Corticosteroids may also be beneficial in the event of underlying adrenal insufficiency. However, findings from retrospective studies are inconsistent. Senda et al (82) found no significant improvement in the prognosis of patients with TS treated with glucocorticoids compared with those treated without glucocorticoids). On the other hand, Furukawa et al (68) reported that patients receiving sufficient glucocorticoid doses had a better prognosis compared to patients with insufficient doses. Corticosteroid therapy may be stopped once the patient is stabilized and if there is no suspicion of accompanying adrenal insufficiency.

##### 3. Blocking thyroid hormone synthesis and secretion

###### Antithyroid drugs

The dose of ATDs administered to patients with TS is typically higher than that required to completely inhibit thyroid hormone synthesis. PTU 500-1000 mg loading dose orally, via nasogastric tube, or per rectum, then 250 mg every 4 hours. The potential advantage of PTU over MMI/CBZ is the additional benefit of peripheral blockade of T4 to T3 conversion and guidelines have traditionally favored PTU for this reason (6). Alternatively, MMI 60-80 mg/day or CBZ 80-100 mg/day may be used, as these appear to have equivalent efficacy in TS compared to PTU. In particular, strongly consider MMI/CBZ rather than PTU if disturbed liver function is present (3× upper limit of normal for bilirubin/liver enzymes). However, Japanese surveys revealed that MMI was preferentially used in TS, and a recent population-based cohort study from the U.S., including almost 1400 patients with TS found no difference in mortality, length of hospitalization, or adverse events between the PTU and MMI group, suggesting that MMI has also a role in TS (83). The dose of ATDs should be lowered as soon as practically possible to PTU 400 mg/day, MMI 30 mg/day, or CBZ 40 mg/day. Antithyroid treatment should be continued until a final decision regarding long-term therapy, surgery, or RAI can be made.

###### Iodine

When administered in large amounts, iodine exerts a dual suppressive effect on the thyroid gland. The Wolff–Chaikoff effect, which refers to inhibition of new hormone synthesis (organification of iodide and coupling), and the Plummer effect which describes inhibition of release of preformed thyroid hormone from the gland. Accordingly, iodine inhibits synthesis (Wolff–Chaikoff effect) and release of thyroid hormones (Plummer effect) (84, 85). Potassium iodide saturated solution (SSKI 5 drops (50 mg iodide/drop [0.05 mL]) orally every 6 hours) or potassium iodide-iodine solution (Lugol's solution 8 drops 4 times a day) should be given 1 hour after the first dose of thionamide, to ensure that any additional iodine load is organified under conditions of blocked hormone synthesis and to avoid providing additional substrate for hormone production (6). Iodine solutions can be locally irritant and must be diluted (eg, in milk or orange juice) before oral/nasogastric administration. Iodide therapy should be stopped once the patient is stabilized. A recent observational study indicated that iodide was not effective in the overall TS cohort, but lower mortality and shorter hospital stay were observed only among patients with GD (86).

##### 4. Adjunctive therapies

Cholestyramine (4 g orally 4 times daily) can be beneficial as it binds thyroid hormones in the gastrointestinal tract, thereby reducing reabsorption and enhancing clearance. Because cholestyramine can also reduce the absorption of many concomitant oral medications, other drugs should be administered at least 4-6 hours apart (87).

Lithium (300 mg every 6 hours and titration to lithium level of 0.8-1.2 mEq/L) can be considered as it inhibits thyroid hormone release from the thyroid (79) but toxicity may be an issue.

In refractory cases or acute liver failure, plasmapheresis may be applied to stabilize patient before salvation thyroidectomy (88).

##### 5. Addressing precipitating factors

Any precipitant must be treated aggressively (eg, broad spectrum intravenous antibiotics for infection).

### Case discussion

Treatment with PTU 600 mg loading dose and thereafter 200 mg every 4 hours was continued for the first 48-72 hours, until the patient's temperature, heart rate, and neurological status had clearly improved, and then tapered over the next several days to a conventional antithyroid maintenance dose, guided by serial thyroid function tests. Propranolol 40 mg every 8 hours was maintained throughout the acute phase to control tachyarrhythmia and was gradually reduced and discontinued once sinus rhythm, heart rate <90 bpm, and hemodynamic stability were consistently achieved. Intravenous hydrocortisone, 100 mg bolus followed by 50 mg every 6 hours was administered for approximately 3-5 days to reduce peripheral T4-to-T3 conversion and cover potential relative adrenal insufficiency and was then tapered and stopped after sustained clinical improvement. Lugol's iodine 5 drops 3 times daily was initiated 1 hour after the first PTU dose and continued for 7-10 days, after which it was discontinued to avoid loss of the acute iodine effect. Cholestyramine 3 gr 3 times daily was administered during the first week to enhance thyroid hormone clearance and was stopped once free hormone levels had substantially declined and the patient had stabilized. Upon further evaluation, TRAb and TPOAb were found positive, establishing the diagnosis of GD. After resolution of the storm, she was transitioned to standard-dose methimazole with close outpatient follow-up, with the goal of maintaining euthyroidism and planning definitive treatment of GD to prevent recurrence. She was counseled that recurrent or uncontrolled thyrotoxicosis is the main risk factor for another episode of thyroid storm and was advised to adhere strictly to antithyroid therapy and seek early medical review during intercurrent illness.

This case illustrates a life-threatening presentation of thyroid storm with predominant neurological and cardiovascular manifestations. The markedly elevated BWPS, together with severe thyrotoxicosis, hyperdynamic goiter and positive thyroidal antibodies, supported the diagnosis of thyroid storm secondary to previously unrecognized GD. SARS-CoV-2 infection likely acted as the precipitating factor, highlighting viral infections as important triggers of thyroid storm. Prompt recognition and early initiation of multimodal therapy were crucial in stabilizing the patient and preventing further deterioration. This case underscores the importance of considering thyroid storm in critically ill patients with unexplained hyperpyrexia, tachyarrhythmia, and altered mental status, particularly in the context of recent viral infection.

## Conclusion

In conclusion, hyperthyroidism is a complex endocrine disorder with the potential to cause severe, multisystem complications such as AF, TPP, and TS. Prompt recognition and individualized management strategies are vital to mitigating morbidity and mortality associated with these conditions. The cases presented highlight the importance of achieving euthyroidism as the cornerstone of treatment, alongside addressing specific complications with appropriate pharmacological and supportive interventions. Despite advancements in understanding, gaps remain in high-quality evidence for the holistic management of hyperthyroidism-related complications, underscoring the need for ongoing research and clinical vigilance.

## Learning points

Hyperthyroidism-related AF

- AF is the most common cardiac condition associated with hyperthyroidism.
- Two-thirds of the patients experience spontaneous return to sinus rhythm after controlling thyroid function, usually within 4-6 months.
- Management of hyperthyroidism-related AF includes ATDs (euthyroidism), beta-blockers (rate control) and anticoagulation based on validated risk stratification tools (CHA2DS2-VASc).

Thyrotoxic periodic paralysis

- TPP is characterized by transient episodes of muscle weakness/paralysis, hypokalemia (due to intracellular shift and not true deficit) and thyrotoxicosis.
- TPP most often affects young men of Asian ancestry.
- Precipitating factors: preceding the TPP attack within 24 hours (high-carbohydrate ingestion, strenuous exercise).
- Caution on potassium supplementation to avoid rebound hyperkalemia.

Thyroid storm

- TS is a clinical diagnosis—clinical judgment is essential.
- Thyroid function testing cannot distinguish TS vs uncomplicated thyrotoxicosis and scoring systems like BWPS and JTA may help clinical judgement.
- Multipillar therapy including ATDs, beta-blockade, glucocorticoids, and management of underlying disorder.

## Data Availability

No primary data were generated for this work. The review is based on previously published literature.

## Abbreviations

ACCP: American College of Chest Physicians
AF: atrial fibrillation
AHA: American Heart Association
ATA: American Thyroid Association
ATDs: antithyroid drugs
BWPS: Burch–Wartofsky Point Score
C_2_HEST: Coronary artery disease, chronic obstructive pulmonary disease, hypertension, systolic heart failure and thyroid disease
CBZ: carbimazole
CHA_2_DS_2_-VASc: congestive heart failure, hypertension, age between 65 and 74 years, female sex, diabetes, and vascular disease, 2 points for age older than 75 years and previous stroke
DOACs: direct oral anticoagulants
ECG: electrocardiogram
ESC: European Society of Cardiology
ETA: European Thyroid Association
FT3: free triiodothyronine
FT4: free thyroxine
GD: Graves’ disease
HAS-BLED: hypertension >160, abnormal renal function; creatinine >2.26 mg/dL, abnormal liver function; cirrhosis or bilirubin >2× normal with AST/ALT/AP >3× normal, stroke, bleeding or predisposition to bleeding, labile INR, age >65, medications predisposing to bleeding, alcohol use
HRS: Heart Rhythm Society
JTA: Japan Thyroid Association
MMI: methimazole
PTU: propylthiouracil
RAI: radioiodine ablation
TPOAb: thyroid peroxidase antibodies
TPP: thyrotoxic periodic paralysis
TRAb: thyrotropin receptor antibodies
TS: thyrotoxic storm
TSH: thyroid-stimulating hormone
